# Surgical Management of Multiple-Level Lumbar Spinal Schwannomas: A Case Report

**DOI:** 10.7759/cureus.41113

**Published:** 2023-06-28

**Authors:** Chukwuyem Ekhator, Ramin Rak

**Affiliations:** 1 Neuro-Oncology, College of Osteopathic Medicine, New York Institute of Technology, Long Island, USA; 2 Neurosurgery, Neurosurgical PC (NSPC) Brain and Spine Surgery, Rockville Center, USA

**Keywords:** benign tumors, spinal cord tumors, extradural schwanomma, benign spinal tumor, schwanomma

## Abstract

The increase in benign spinal tumors among adults over the last decade has been a major cause of concern. This worrisome trend has been attributed to many factors, including improved detection techniques, enhanced access to medical care, and the aging population. The research primarily focuses on Schwannoma, which is a rare type of tumor that arises from Schwann cells, which are responsible for producing the myelin sheath that surrounds and protects nerves. Although the majority of schwannomas are benign, there have been instances where they have transformed into malignant tumors, potentially leading to significant morbidity and mortality. We report a case of a 68-year-old woman who presented with progressive back pain and weakness in both lower limbs over the past months. The pain was initially localized to the lower back but gradually intensified and radiated down to the legs. The patient reported difficulty walking and a sensation of tingling and numbness in the feet. She denied any recent trauma or significant medical history. On physical examination, there was reduced muscle strength (3/5) in both lower limbs. The patient exhibited hyporeflexia in the knees and ankle. A magnetic resonance imaging (MRI) of the spine was performed, revealing a well-defined mass lesion located in the lumbar region, compressing the spinal cord from L2 to L5. The patient was counseled and prepared for surgical resection of the tumor. Histopathological findings revealed features of peripheral nerve sheath tumors and cellular schwannomas. The patient recovered well postoperatively. The surgeon operating should be mindful of the potential presence of a mobile schwannoma, even though it is rarely mentioned in the literature. Being aware of this possibility can help prevent unnecessary surgical dissection, which can lead to higher rates of complications and morbidity. Although it is plausible that this case could have involved a mobile schwannoma, there was not enough evidence to support it as we performed a laminectomy on multiple levels due to the tumor's size.

## Introduction

Spinal tumors are relatively rare, with the most common types including meningiomas, schwannomas, and chordomas. Among these, schwannomas are the least common, accounting for approximately 7% to 23% of all benign spinal tumors [1,2]. Schwannomas are typically solitary, encapsulated tumors that arise from Schwann cells of the peripheral nervous system [3]. While schwannomas can occur anywhere in the body where Schwann cells are present, they are most commonly found in the head, neck, and spinal cord regions [4]. Spinal schwannomas are typically slow-growing tumors that arise from the dorsal roots of spinal nerves and can be intradural or extradural. Extradural schwannomas are more common, accounting for approximately 60% to 70% of cases, and typically present as painless masses that compress adjacent structures [5,6]. Intradural schwannomas are less common and can cause a variety of symptoms, depending on their location [7]. Schwannomas can occur at any age, but they are most commonly diagnosed in adults aged between 30 and 50 years [2,8]. There is no clear gender predilection for spinal schwannomas [9]. The diagnosis of spinal schwannoma is typically conducted using a combination of imaging studies, including magnetic resonance imaging (MRI) and computed tomography (CT) scans. These studies can help to determine the location and size of the tumor, as well as its relationship to adjacent structures [10]. In some cases, a biopsy may be necessary to confirm the diagnosis [11]. The treatment of spinal schwannoma depends on a variety of factors, including the size and location of the tumor, as well as the patient's age and overall health. In some cases, observation may be appropriate, particularly for small tumors that are not causing symptoms [12]. For larger tumors, surgical resection is typically the treatment of choice. While complete resection of the tumor can be curative, in some cases, subtotal resection may be necessary due to the location of the tumor [13]. In cases where surgery is not feasible or the patient is not a candidate for surgery, radiation therapy may be used [14]. Spinal schwannomas are rare and can cause significant morbidity. Reporting cases of schwannoma can also help to identify potential risk factors associated with the development of these tumors and improve treatment outcomes for patients. This literature review and case report takes a comprehensive approach to understanding schwannomas and aim to identify risk factors, early warning signs, and effective treatments that can lead to improved outcomes for patients. Key resources such as PubMed, the National Center for Biotechnology Information (NCBI), and PubMed Central (PMC) have been instrumental in consolidating and strengthening the research findings

## Case presentation

Structure of spinal tumors

A spinal tumor is typically categorized according to its location within or outside the dura mater and in or out of the medulla or spinal cord substance. Numerous kinds of tumors exhibit distinct behaviors and necessitate various therapies. Various types of tumors usually behave differently and also require different treatment approaches. Extradural tumors are those that are external to the dura mater or are outside of the spinal cord. They are universal. The vertebrae are where these cancers typically develop. Vertebral column tumors are cancers of the vertebral column. The spinal cord's covering, the dura, is absent from the area where the tumor is situated. This location's frequency of occurrence is about 55% higher. The lesions are frequently linked to metastatic cancer or, less frequently, to schwannomas that develop from the nerve root covering cells. An extradural tumor can occasionally penetrate the intervertebral foramina and lie partially inside and partially outside the spinal canal. In Intradural-extramedullary, the tumor is situated outside the genuine spinal cord but inside the delicate dura, which covers the spinal cord. In this area, occurrences occur 40% of the time.

Case presentation

We present a case of a 68-year-old woman who presented with progressive back pain and weakness in both lower limbs over the past months. The pain was initially localized to the lower back but has gradually intensified and radiated down to the legs. The patient reported difficulty walking and a sensation of tingling and numbness in the feet. She denied any recent trauma or significant medical history. On physical examination, there was reduced muscle strength (3/5) in both lower limbs. The patient exhibited hyporeflexia in the knees and ankle. Sensory examination revealed decreased sensation to light touch and pinprick in the lower extremities, extending below the knees. The patient had intact upper limb strength and sensation, and cranial nerve examination was unremarkable. An MRI of the spine was performed, revealing a well-defined mass lesion located in the lumbar region, compressing the spinal cord from L2 to L5. Spinal canal narrowing and lumbar spinal stenosis compressed the nerves that run through the lower back and into the legs [15-27]. The spinal canal typically narrows gradually over many years or decades. With time, the disks lose some of their elasticity, which lowers their height and may lead to the bulging of a hardened disk into the spinal canal. Additionally, ligaments may stiffen and bone spurs may develop. This may or may not cause symptoms but can all contribute to central canal narrowing [19]. Inflammation, nerve(s) compression, or both may cause symptoms. In this case, further investigation with imaging was necessary to localize the cause of nerve impingement. The patient was counseled and prepared for surgical resection.

The interoperative phase of the study

**Figure 1:**
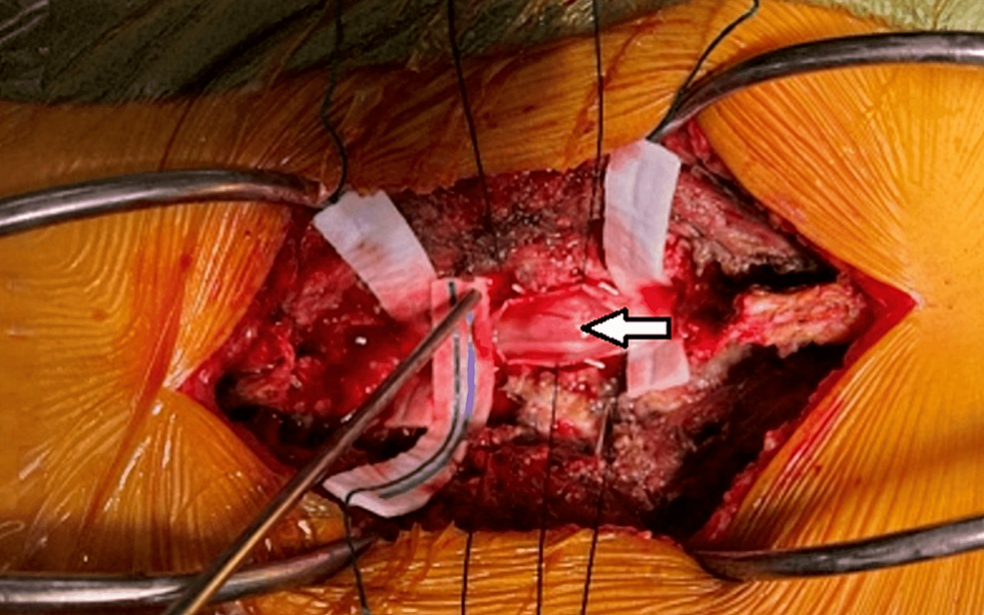
Exposure of the surgical site after resection of spinal tumor (white arrow). Image used with permission from the patient.

The surgical site was located, and the incision was marked with a marking pen on the posterior aspect of the midline inside the lumbar spine region utilizing preoperative X-rays and fluoroscopy. The surgery site was then prepared and wrapped sterilely as normal. Steroids were administered after the issue was discussed with the anesthesia staff before surgery to maintain the average arterial pressure over 85. The surgical area was then prepared and wrapped sterilely as normal (Figure 1). After a sufficient break, the incision was made through the skin and into the subcutaneous tissues using a 10-blade scalpel. Bovie electrocautery ( Symmetry Surgical Inc., Antioch, TN, USA) was used to further separate the tissues up to the fascia, and it was combined with Aquamantys (Medtronic, Dublin, Ireland) to achieve hemostasis.

**Figure 2:**
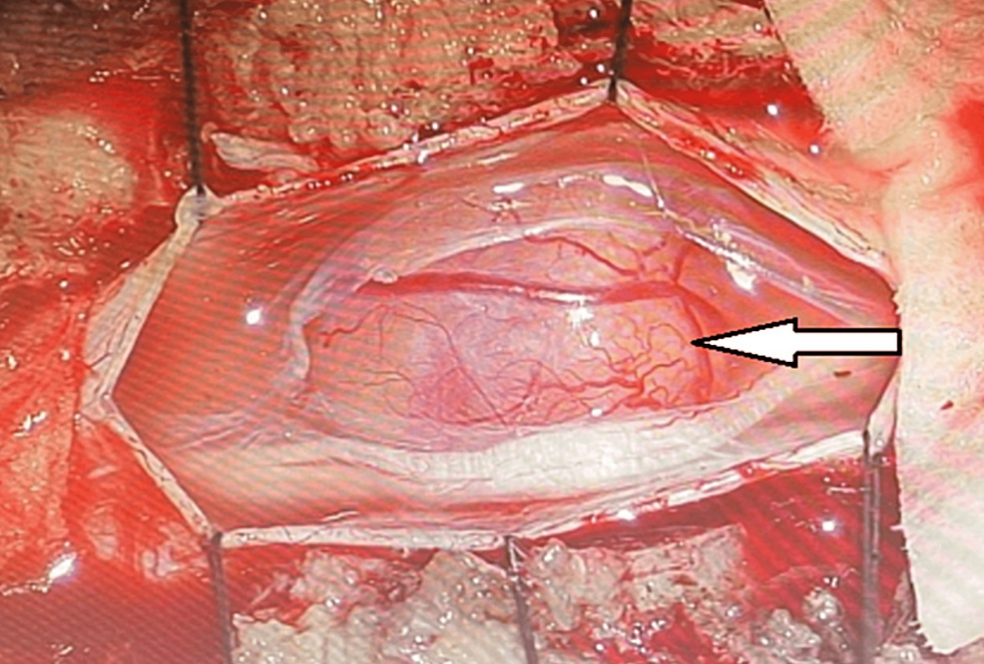
Exposure and excision of spinal tumor (white arrow). Image used with permission from the patient.

**Figure 3:**
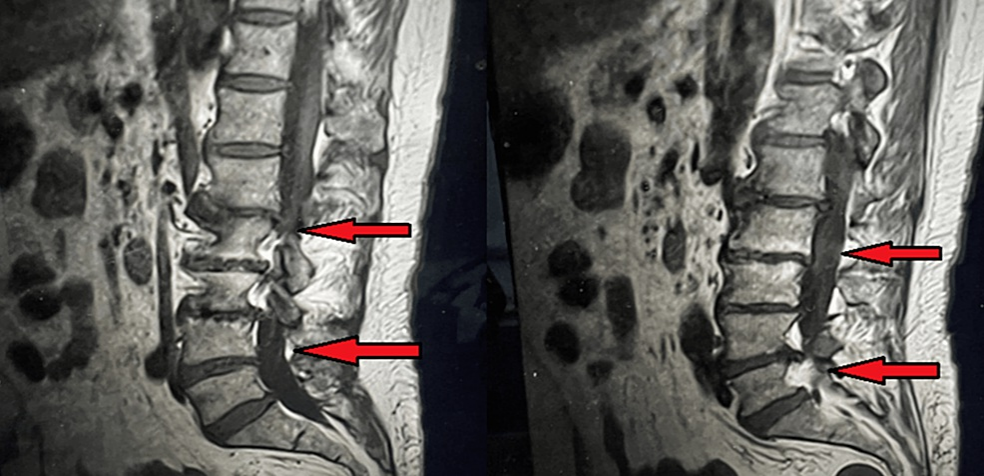
Benign spinal tumor with pressure on L2 to L5. (A) Left (red arrow) before resection of the mass with pressure on L2 to L5. (B) Right (red arrow) after resection of the mass with relief of pressure from L2 to L5. Images are original and were used with permission from the patient.

The fascia was divided over the midline spinous processes after retraction was applied (Figure 1). At the region of the periosteum, the spinous process and laminas were separated from the paraspinal muscles (Figure 2). Retractors were used that were deeper. The level of the surgery was localized using further fluoroscopy. Throughout the process, the facet joints, as well as the pars, were all shielded. The laminectomy and deflation between L2 and L5 were then carried out. After properly dissecting the ligamentum flavum, the laminas, and the midline components, they were typically extracted from the thecal sac using a Misonix bone cutter. The laminas were then dissected medially to facets from L2 to L5 (Figure 3).

At all stages from L2 to L5, the canals and the peripheral nerves were under a sizable amount of pressure. The nerve roots were neutralized circumferentially from L2 to L5, bilaterally, with great care, loupe magnification, and expert surgical skill. The nerve roots and all foramina were fully unobstructed by soft or arthritic tissues. The intradural excision of the tumor was then carried out. The dural resection was carried out utilizing microsurgical methods and microsurgical instruments under the supervision of a surgical microscope. The first incision on the thecal sac's midline was performed with a 15-blade scalpel. Woodson was then inserted, and the thecal sac was further dissected with a durotomy, mostly on the centerline from the bottom section of L1 to L3. We provided adequate exposure to the thecal sac to promote the restoration of the dura following tumor excision. The arachnoid layer was removed, cerebrospinal fluid was drained, and the tumor was visible as soon as the dura was exposed.

To access the tumor capsule from a nonstimulating area during the surgery, intraoperative neuromuscular stimulation and electrodes were used to activate the tumor. The aim was to preserve nerve function as much as possible. By using intraoperative activation and monitoring, it became evident under the operating microscope that the tumor originated from the nerve root. Using 4-0 Nurolon (Ethicon, Somerville, NJ, USA) stitches, the dura's lateral margins were kept retracted. With the use of bipolar electrocautery and microscissors, the tumor capsule was delicately separated. The tumor was delicately debulked with Rhoton microsurgical tools.

**Figure 4:**
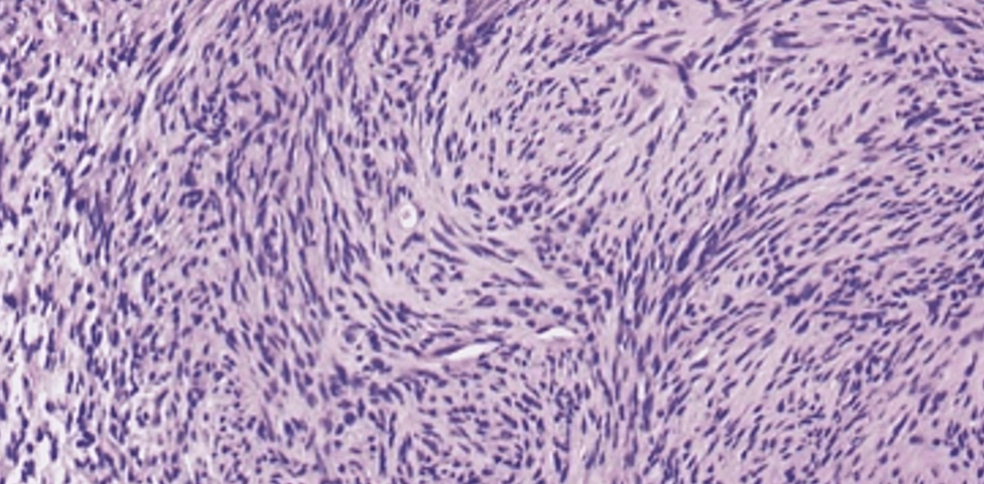
Cellular schwannoma demonstrating numerous whorls. Referenced from morphologic and immunohistochemical features of peripheral nerve sheath tumors and cellular schwannomas [19].

Pathology received a portion of the tumor as a frozen specimen (Figure 4). To the greatest extent possible, the nerve root activity was preserved by further dissecting the tumor inside the capsule. The tumor was removed, and the permanent specimens were submitted to pathology. A consultation with intraoperative pathology revealed that the frozen section was compatible with what was mostly a benign schwannoma. Surgiflo and thrombin were used to achieve hemostasis further, and irrigation was used to remove all bleeding from the thecal sac and subarachnoid area. The tumor was successfully decompressed and removed, and once the tumor was removed, all motor stimulations and nerve functions were unaffected. We applied more watering after that and then closed the dura. First, the dura was stitched shut using 4-0 Nurolon sutures, which were also used during the durotomy to maintain exposure. The dura was then stitched shut using a 6 mm running Prolene stitch as a second layer of closure. At this point, the anesthesia team performed a Valsalva maneuver, and no evidence of cerebrospinal fluid leakage through the dura closure was found. Gelfoam, thrombin, Surgiflo, and thrombin were used for further irrigation and hemostasis. After thorough irrigation, a layer of DuraGen was placed over the durotomy area to cover the dura. Finally, DuraSeal was applied to properly seal off the area. The dura, the dura sealant, and the dura closure were all outstanding. The patient did well during the treatment and had no difficulties during the surgery. About 50 mL of blood was thought to have been lost during the treatment. Following surgery, the patient was placed in the supine position in a hospital bed, extubated, and taken in a stable state to the recovery area. The patient recovered well postoperatively.

## Discussion

Differential diagnosis

Primary spinal tumors have no recognized cause. However, individuals with weakened immune systems are more susceptible to developing lymphomas of the spinal cord, which involve abnormal growth of immune cells known as lymphocytes. There is indeed a hereditary component observed in certain families that appears to be associated with a higher incidence of spine cancers [15]. The most typical symptom of benign tumors is nonmechanical back discomfort, particularly in the middle to lower back. However, the pain could get better at night when lying down and get worse with movement. Even when conservative, nonsurgical treatments, which can frequently help relieve back pain due to mechanical causes, are used, discomfort may move further than the spine to the hips, legs, feet, or arms and may worsen with time. Other symptoms may appear depending on the nature and location of the tumor, mainly as it grows and presses against the spinal cord, nerve roots, blood vessels, or vertebral bones in the spine. Schwannomas can cause various symptoms depending on their location and size. Spinal schwannomas can cause various symptoms such as pain, weakness, tingling sensations, discomfort, or paralysis. 

Meningiomas, schwannomas, and neurofibromas can compress the root of the spinal cord and anywhere along the filum terminale. Ependymoma tumors may develop intramedullary. They typically come via glial or ependymal cells, which are present throughout the spinal cord's interstitium. In this area, the frequency of occurrence is about 5%. In addition, metastasis also accounts for some spinal tumors. The spinal column is where bone metastases occur most frequently. According to estimates, cancer spreads to the spine in 50% of cancer patients. Vertebral hemangiomas, which develop in the bony spine, are the most typical primary spine tumors. Since these lesions are benign, symptoms like pain are seldom. Pollack et al. [16] conferred that astrocytomas are the most frequent tumors in children and the second most frequent in adults. According to Sturm et al. [17], research on spinal astrocytomas is still in its early stages due to the rarity of this type of tumor, limited availability of tissue for study purposes due to small tumor size, and the associated risks of complications with surgical resection of infiltrative spinal cord tumors. As a result, genomic studies specifically focused on spinal astrocytomas are currently in their early stages of development. Such dangers include death, paralysis, progressive neurological deficiency, and bowel and bladder dysfunction [18-22] 

Strengths and weaknesses

The surgeon operating should be mindful of the potential presence of a mobile schwannoma, even though it is rarely mentioned in the literature. Being aware of this possibility can help prevent unnecessary surgical dissection, which can lead to higher rates of complications and morbidity. Although it is plausible that this case could have involved a mobile schwannoma, there isn't enough evidence to support it as we performed a laminectomy on multiple levels due to the tumor's size. For better surgical outcomes, various measures can be taken to reduce these complications, such as adopting perioperative imaging techniques and intraoperative sonography. In most cases, spinal schwannomas are successfully removed with surgery. The prognosis is generally excellent or stable in cases where there has been prior subtotal removal of the tumor, despite the potential risk of recurrence.

## Conclusions

Removal of the schwannoma typically results in improved peripheral nerve function with minimal risk of new postoperative neurological abnormalities. The case presented in this literature review suggests that the patient had a high likelihood of making a full recovery. In most cases, postoperative histological examination is used to diagnose schwannomas, even in tiny nerve branches. Sensory abnormalities following surgery usually resolve completely or require only minor treatment without the need for drugs or nerve blocks. Benign spinal tumors are relatively rare, and reporting cases of schwannoma will help to establish an in-depth understanding of the disease process and improve the treatment strategies. 
